# The Effects of Quercetin Supplementation on Eccentric Exercise-Induced Muscle Damage

**DOI:** 10.3390/nu11010205

**Published:** 2019-01-21

**Authors:** Ilenia Bazzucchi, Federica Patrizio, Roberta Ceci, Guglielmo Duranti, Paolo Sgrò, Stefania Sabatini, Luigi Di Luigi, Massimo Sacchetti, Francesco Felici

**Affiliations:** 1 Laboratory of Exercise Physiology—Department of Movement, Human and Health Sciences, Università degli Studi di Roma “Foro Italico”, Piazza Lauro De Bosis 6, 00135 Roma, Italy; federica.patrizio@libero.it (F.P.); massimo.sacchetti@uniroma4.it (M.S.); francesco.felici@uniroma4.it (F.F.); 2 Laboratory of Biochemistry of Movement—Department of Movement, Human and Health Sciences, Università degli Studi di Roma “Foro Italico”, Piazza Lauro De Bosis 6, 00135 Roma, Italy; roberta.ceci@uniroma4.it (R.C.); guglielmo.duranti@uniroma4.it (G.D.); stefania.sabatini@uniroma4.it (S.S.); 3 Endocrinology Unit—Department of Movement, Human and Health Sciences, Università degli Studi di Roma “Foro Italico”, Piazza Lauro De Bosis 6, 00135 Roma, Italy; paolo.sgro@uniroma4.it (P.S.); luigi.diluigi@uniroma4.it (L.D.L.)

**Keywords:** muscle damage, muscle weakness, electromyography

## Abstract

The aim of the present investigation was to test the hypothesis that quercetin (Q) may prevent the strength loss and neuromuscular impairment associated with eccentric exercise-induced muscle damage (EEIMD). Twelve young men (26.1 ± 3.1 years) ingested either Q (1000 mg/day) or placebo (PLA) for 14 days using a randomized, double-blind, crossover study design. Participants completed a comprehensive neuromuscular (NM) evaluation before, during and after an eccentric protocol able to induce a severe muscle damage (10 sets of 10 maximal lengthening contractions). The NM evaluation comprised maximal voluntary isometric contraction (MVIC) and force–velocity relationship assessments with simultaneous recording of electromyographic signals (EMG) from the elbow flexor muscles. Soreness, resting arm angle, arm circumference, plasma creatine kinase (CK) and lactate dehydrogenase (LDH) were also assessed. Q supplementation significantly increased the isometric strength recorded during MVIC compared to baseline (+4.7%, *p* < 0.05). Moreover, the torque and muscle fiber conduction velocity (MFCV) decay recorded during the eccentric exercise was significant lower in Q compared to PLA. Immediately after the EEIMD, isometric strength, the force–velocity relationship and MFCV were significantly lower when participants were given PLA rather than Q. Fourteen days of Q supplementation seems able to attenuate the severity of muscle weakness caused by eccentric-induced myofibrillar disruption and sarcolemmal action potential propagation impairment.

## 1. Introduction

Eccentric muscle action is characterized by the lengthening of skeletal muscle while producing force. This mechanical stress induces breaking of the contractile components of muscle tissue followed by a subsequent inflammatory phase that is responsible for secondary muscle damage [1]. The acute phase is characterized by an impaired action potential propagation along the sarcolemma which is responsible for an immediate decrease in muscle strength. This is a direct indicator of an impairment in the neuromuscular efficiency in the early stages of eccentric exercise-induced muscle damage (EEIMD). 

Eccentric exercises are an essential part of athletes’ training programs, at both the professional and recreational levels. However, the discomfort and impairment in the quality of performance caused by EEIMD induce athletes to search for strategies to prevent/alleviate those symptoms. Fruit juices that are known to be particularly rich in vitamin C and E, flavonoids, carotenoids and most antioxidant molecules consumed in pills are among the most commonly used sport supplements [2,3] to protect against damage and from reactive oxygen species (ROS) formed during exercise. Unfortunately, this strategy has been only corroborated by a few results, and the ability of antioxidant vitamins to reduce EEIMD is still under debate. Recently the attention has shifted to nutraceutical bioactive compounds, and quercetin (Q), a flavonol-type polyphenol, has been demonstrated to exert a variety of bioactive effects that are related to its strong antioxidant and anti-inflammatory properties [4].

For this reason, the use of Q to reduce EEIMD seems plausible, although few studies have focused on the validation of this hypothesis and have focused mainly on the effect of Q supplementation on muscle damage induced by the oxidative stress derived from prolonged exercise (i.e., cycling, treadmill exercise), reporting a modest or no effect on its prevention [5,6]. Interestingly, a recent study from our group provided evidence that Q supplementation makes erythrocytes more able to cope with oxidative insult following eccentric exercise [7]. However, to the best of our knowledge, no studies have investigated the effect of Q supplementation on skeletal muscle electrophysiological modifications induced by eccentric exercise.

In agreement, the primary objective of the present study was to test the hypothesis that a chronic Q supplementation may prevent the strength loss and electromyographic (EMG) alterations that occur during eccentric exercise. To this end, a comprehensive neuromuscular evaluation was conducted before, during and after an eccentric protocol able to induce severe muscular damage.

## 2. Materials and Methods

### 2.1. Participants

Twelve moderately active men (age 26.1 ± 3.1 years; body mass 75.1 ± 7.1 kg; stature 1.79 ± 0.04 m) were recruited. The exclusion criteria included: a) history or signs of metabolic, renal or neurological diseases; b) usage of dietary supplements with vitamins and/or antioxidants for at least 6 months prior to the study; and c) undertaking regular resistance training within six months of the study starting date. For the duration of the study, participants were asked to continue their habitual exercise and nutritional routines. In addition, to avoid the ingestion of foods containing Q and other antioxidant properties, from 1 week before and during the study period, nutritional supplements and ergogenic aids were not allowed. Subjects were also asked to refrain from taking anti-inflammatory medications while taking part in the study. Participants were fully informed of the experimental protocol and the possible risks of the investigation before giving their written informed consent. The Ethics Committee of the University of Rome “Sapienza” approved the protocol (process number 2621/15).

### 2.2. Experimental Overview

This study used a double-blind, placebo-controlled, randomized crossover design. Volunteers were randomized to quercetin (Q) or placebo (PLA) treatment and then crossed over to the opposite condition after a 3-week washout period [8]. On the first visit, volunteers were familiarized with the experimental procedures. The neuromuscular (NM) evaluation was performed on an upper limb. The arm exposed to the eccentric protocol (right (R) or left (L)) was reversed when the protocol was repeated with the other treatment. The association between treatment (Q or PLA) and arm (R or L) was also randomly assigned so that the limb dominance was not uniquely associated with the supplementation.

**Figure 1:**
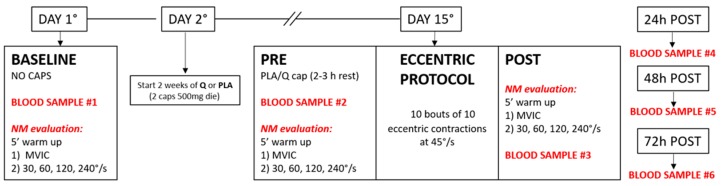
Flowchart of the experimental protocol. NM: neuromuscular.

NM tests and other indices of muscle damage (soreness, elbow angle, arm circumference) were assessed before starting the treatment (BASELINE), after 14 days of Q/PLA intake (PRE) and immediately after the eccentric protocol (POST) (Figure 1).

### 2.3. Treatment

During the first session, volunteers underwent a complete pre-intervention evaluation (BASELINE). Then, they were randomly assigned to Q or PLA. Starting from the following day, for 14 consecutive days, participants ingested 2 capsules containing 500 mg of quercetin aglycone in crystalline powder (Farmalabor Srl, Milan, Italy), one at breakfast and one 12 hours later, to achieve a daily experimental dose of 1000 mg [9]. Placebo capsules were indistinguishable by taste and appearance from those of Q. Volunteer compliance was monitored daily by the investigators. 

### 2.4. NM Evaluation

Elbow flexion torque was measured with an isokinetic dynamometer (Kin-Com, Chattanooga, TN, USA) as previously described [10]. The surface EMG signals were recorded with a linear array of 4 electrodes (10 mm interelectrode distance; OTBioelettronica, Turin, Italy) from the biceps brachii muscle (BB). The optimal position and orientation of the electrodes were determined as previously described [10]. EMG signals were amplified, band-pass filtered (10–450 Hz; EMG USB2+, OTBioelettronica, Turin, Italy) and sampled at 2048 Hz. 

After a standardized warm-up, the following NM tests were assessed: 1) maximal voluntary isometric contractions (MVIC); 2) force–velocity relationship task (FV).

1 MVIC: The joint angle was fixed at 90° (0°, full extension). The task consisted of rapidly increasing the force “as hard as possible” to produce a maximal contraction with the elbow flexors. All participants were verbally encouraged to exceed a visual target and to maintain the MVIC for 2–3 s before relaxing. Three attempts separated by 5 min were performed.
2 FV: After the MVIC, the force–velocity curve was assessed. Participants performed a set of 3 maximal isokinetic back-to-back elbow flexions at 30, 60, 120, and 240°/s with a 5 min-rest [11,12]. The range of motion (ROM) was 100° starting from 40° to 140°.

### 2.5. Eccentric Protocol

The dynamometer was set to an angular velocity of 45 °/s and participants were instructed to resist maximally during the whole ROM (from 40° to 140°). Each participant completed 10 bouts (separated by a 30 s-rest) of 10 maximal lengthening contractions of the elbow flexors. Each eccentric contraction lasted 2 s followed by a 6-s rest in which the dynamometer arm returned automatically backward as previously described [13]. 

### 2.6. Indices of EEIMD

Pain intensity was assessed with a visual analogue scale (VAS) in response to the question “Indicate on the scale below the intensity of the pain in your arm”. The 10-cm VAS was anchored at left with “no pain” (score of 0) and at right with “worst pain imaginable” (score of 10) [14]. The relaxed elbow angle was measured using a goniometer while participants stood upright with their arm damaged relaxed by their side. Anatomical landmarks were located at the midpoint between the acromion and the coracoid process, the lateral epicondyle, and the midpoint of the ulnar and radius styloid processes. These points were marked at the beginning of the experiment to ensure reliable measurements. A decrease in elbow joint angle has been shown to be indicative of an exercise-induced rise in the passive tension of the elbow flexor muscles [15].

### 2.7. Blood Samples Collection

Blood samples (10 mL for each draw) were collected from the forearm vein following the draw chart in Figure 1. They were maintained at +4 °C, and plasma was immediately separated and stored at −80 °C until biochemical assays. All analyses performed on these samples were performed twice in triplicate. Inter- and intra-assays were performed to assess precision within and between assays. Since the delayed onset of the increase in plasma markers after an eccentric induced muscular damage is well known, we also collected blood samples at 24 h, 48 h and 72 h after the eccentric protocol.

### 2.8. Data Analysis

#### 2.8.1. NM Data

All data collected during the experiments were analyzed offline (OTBiolab software, OTBioelettronica, Turin, Italy). Maximal Torque and mean fiber conduction velocity (MFCV) were the parameters of interest. MFCV values were estimated on the EMG signals from the two double differentials by means of the cross-correlation technique, as previously described [11,12]. For the eccentric protocol, the maximal torque and MFCV data of the first two repetitions (reps) were averaged and expressed for each of the 10 sets. Moreover, the difference between the average value of the first two reps of the first set and the average value of the last two reps of the tenth set was calculated for torque and MFCV (ΔTorque1st–10th and ΔMFCV1st–10th, respectively). All percentage data (%Torque and %MFCV) are expressed with respect to the maximal value obtained during the MVIC at BASELINE. 

#### 2.8.2. Biochemical Analyses

All chemical reagents were purchased from Sigma-Aldrich Chemical (St. Louis, MO, USA). Plasma creatine kinase (CK) activity was determined spectrophotometrically, according to manufactory recommendations, by a manual procedure using a commercial test kit (Greiner Diagnostic GmbH, Bahlingen, Gremany). Plasma lactate dehydrogenase (LDH) activity was determined spectrophotometrically by quantifying the reduction of NAD+ (measured at 340 nm) at 30 °C in an assay mixture containing 0.2 M Tris-HCl (pH 7.6), 7 mM oxidized NAD+, and 55 mM lactate with a 20 μL sample. The extinction coefficient of NADH at 340 nm is 6.22 mM^−1^ × cm^−1^. One unit of enzymatic activity was defined as the amount of enzyme that forms 1 μmol of product per minute [16].

### 2.9. Statistical Analysis

Statistical analyses were conducted using IBM SPSS Statistics 27 (SPSS Inc, Chicago, Illinois, USA). A two-way repeated measure analysis of variance (RMANOVA) was used to assess differences in all dependent variables measured during the MVIC for the two treatments (PLA, Q) at the 3 time points (BASELINE, PRE, POST). A two-way RMANOVA (treatment (Q, PLA) × set (10 sets)) was used to analyze the effect of treatment on the electromechanical variables (Torque and MFCV) as a function of the sets of the eccentric protocol. Finally, a three-way RMANOVA (treatment (Q, PLA) × time (BASELINE; PRE; POST) × 5 angular velocities (0, 30, 60, 120 and 240°/s)) was used to assess differences in the electromechanical variables as a function of time points during the FV relationship task. When the sphericity assumption was violated, the Greenhouse–Geisser adjustment was performed. In case of significant interactions, a one-way RMANOVA was used with subsequent follow-up Bonferroni tests performed in cases with significant simple main effects. A *p* value < 0.05 was considered statistically significant in all analyses. An a priori analysis was used to determine a sample size that yielded a power value of 0.80 or greater. The results are expressed as means (±SE). 

## 3. Results

### 3.1. Eccentric Protocol

**Figure 2:**
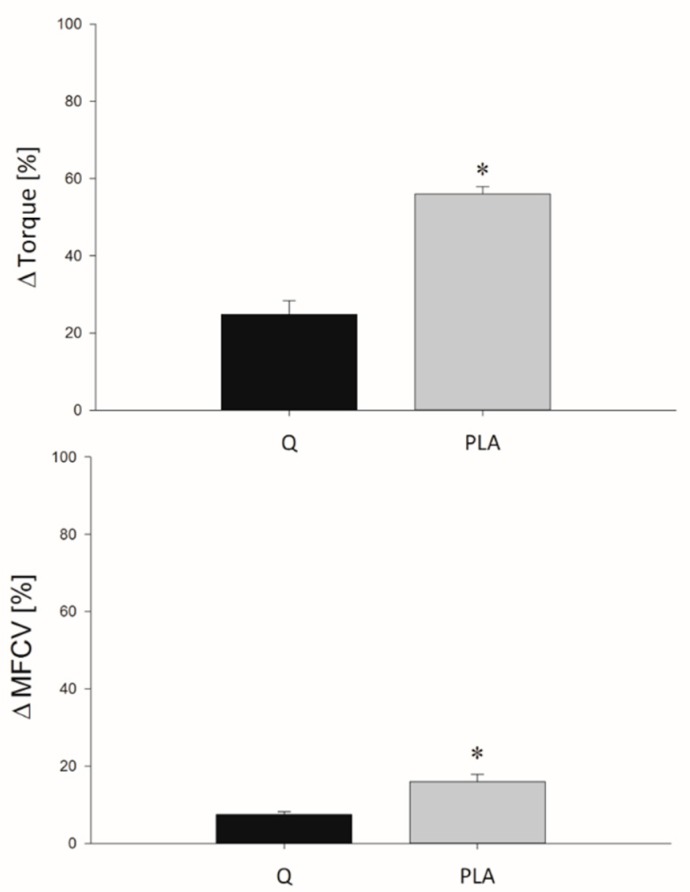
Mean ΔTorque (upper panel) and ΔMFCV values (lower panel) obtained at the end of the 10 sets of eccentric exercise (expressed as a percentage of the initial values) in Q (black bars) and PLA (grey bars) conditions. * *p* < 0.05; significantly different from Q.

ΔTorque and ΔMFCV values obtained during the eccentric protocol are shown in Figure 2. The %Torque decay obtained at the end of the 10 series of eccentric contractions was significantly lower after 14 days of consumption of Q with respect to PLA (*p* < 0.001). The same behavior was found for MFCV values, which showed lower ΔMFCV in Q compared to the PLA condition (*p* < 0.001).

### 3.2. MVIC

**Figure 3:**
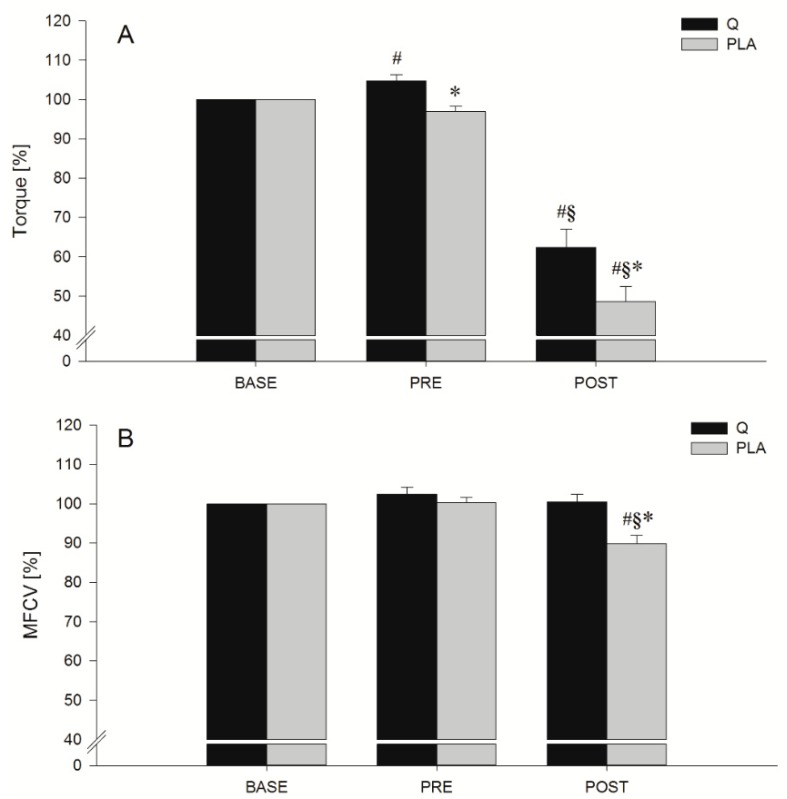
Mean torque and mean fiber conduction velocity (MFCV) values obtained during the maximal voluntary isometric contraction (MVIC) tests before (MVICPRE) and after (MVICPOST) the eccentric exercise in quercetin (Q; black bars) and placebo (PLA; grey bars) conditions. Data are expressed as a percentage of MVICBASE. #, §, * *p* < 0.05; significantly different from baseline (BASE), before (PRE) and Q respectively.

Maximal torque and MFCV values obtained during the MVIC tests are shown in Figure 3. Significant effects of treatment and time were found for both torque (*p* = 0.009 and *p* < 0.001, respectively) and MFCV (*p* = 0.025 and *p* < 0.001 respectively) together with a treatment × time interaction (*p* = 0.033 for torque and *p* = 0.005 for MFCV). Post hoc comparisons showed a significant difference between torque values obtained after the 14 days of consumption of Q or PLA before the eccentric protocol (MVICPRE, *p* = 0.004) and after the eccentric protocol (MVICPOST, *p* = 0.026). Concerning the MFCV, PLA and Q conditions were significantly different only at MVICPOST (*p* = 0.007), showing a significant MFCV decrease after the eccentric protocol when participants consumed PLA. Moreover, there was a notable significantly greater %Torque at MVICPRE compared to MVICBASE (*p* = 0.013) in the Q condition.

### 3.3. FV Relationship Task

**Figure 4:**
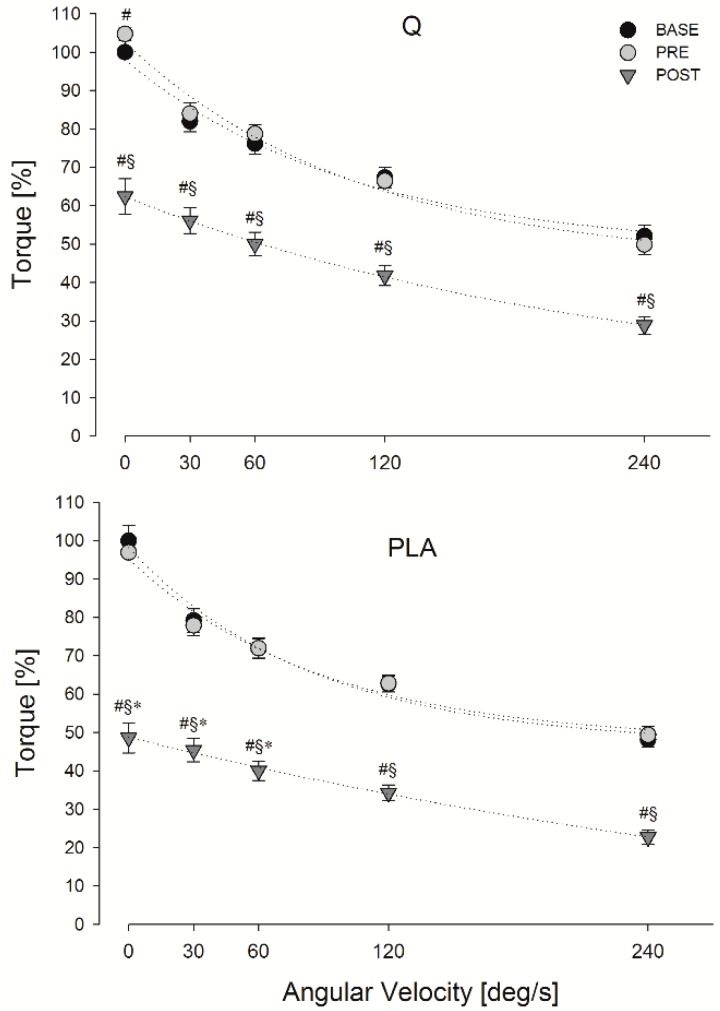
Mean torque values obtained during the FV relationship task in Q (panel A) and PLA (panel B) conditions at baseline (BASE), before (PRE) and after (POST) the eccentric exercise. Data are expressed as a percentage of MVICBASE. #, §, * *p* < 0.05; significantly different from BASE, PRE and Q respectively.

The maximal torque and MFCV values obtained during the FV relationship task in Q and PLA conditions are shown in Figure 4 and Table 1.

Significant main effects of treatment, time point and angular velocity were found for both torque (*p* = 0.008, *p* < 0.001, *p* < 0.001, respectively) and MFCV (*p* = 0.012, *p* < 0.001, *p* < 0.001, respectively). Moreover, a significant treatment, time point and angular velocity interaction was found for torque (*p* = 0.036) and MFCV (*p* = 0.020). Post hoc comparisons showed a significant higher torque decay after the eccentric protocol (POST) when participants consumed PLA compared to Q (*p* = 0.027 at 0°/s, *p* = 0.041 at 30°/s, *p* = 0.028 at 60°/s) (Figure 2). Moreover, the area under the torque curve computed after the eccentric protocol (POST) was significantly lower in PLA vs. Q (*p* = 0.031). Concerning the MFCV, post hoc comparisons showed a significant higher MFCV decay in POST when participants ingested PLA compared to Q (*p* = 0.007 at 0 °/s, *p* = 0.045 at 60 °/s, *p* = 0.006 at 120 °/s, *p* = 0.001 at 240 °/s) (Table 1).

### 3.4. Indices of EEIMD

Mean values of arm circumference, elbow angle and VAS are reported in Table 2. A main effect of time (BASE, PRE, POST) was found for all of the above-mentioned parameters. Post hoc comparisons showed that after the eccentric protocol (POST), arm circumference was significantly (*p* < 0.001) greater both in Q and PLA with respect to BASE and PRE conditions, without a significant main effect of treatment or interaction between treatment and time. The mean VAS values reported by the participants after the eccentric protocol were significantly higher than the values reported in BASE (*p* = 0.017) and PRE (*p* = 0.017) only when the volunteers assumed PLA. In POST, the arm was significantly (*p* < 0.001) more flexed in a relaxed condition in both Q and PLA with respect to BASE and PRE, as revealed by the greater elbow angle (being 0° full extension). Since a treatment x time interaction was found, the post hoc comparison showed also that the arm was more flexed in PLA with respect to Q (*p* = 0.034) only in POST.

Table 3 shows the mean values of %CK and %LDH. As already mentioned in the Methods section, blood samples for the analysis of these two parameters were collected 24 h, 48 h and 72 h after the eccentric protocol. A main effect of time (BASE, PRE, POST, 24 h, 48 h, 72 h), a main effect of treatment (Q vs. PLA) and a treatment × time interaction were found for both parameters. As expected, post hoc comparisons showed that %CK values were different between Q and PLA condition only after 48 h and 72 h (*p* = 0.047 and *p* = 0.026, respectively). Concerning the %LDH, the post hoc comparison revealed significant greater levels in PLA with respect to Q condition at 24 h, 48 h and 72 h after the EEIMD (*p* < 0.001 at any time point).

## 4. Discussion

The primary objective of this study was to investigate the effect of 14 days of supplementation with Q on the prevention of the neuromuscular function impairment caused by acute EEIMD.

The main findings of the present study are as follows: 1) after 14 days of Q supplementation, participants showed a significant increase in the MVIC with respect to baseline; 2) when participants consumed Q, the force and MFCV decay recorded during the eccentric exercise were significant lower than in PLA; 3) MVIC and the force–velocity relationship post EEIMD were significantly lower when participants ingested PLA and this was accompanied by a reduction of MFCV; 4) biochemical and functional indices of muscle damage showed different behaviors after the EEIMD between Q and PLA conditions. 

The significant increase in MVIC after the 14 days of Q supplementation represents an unexpected finding, and it shows, for the first time, an ergogenic effect of Q on neuromuscular function. Several human studies attempted to evaluate Q ergogenic potential in endurance performance and maximal aerobic capacity [5,6,17,18,19]. Those studies have been meta-analyzed [20,21], and whilst Kressler and colleagues concluded that Q supplementation “provides a statistically significant benefit in VO_2max_ and endurance exercise performance”, Pelletier et al. concluded that Q supplementation “is unlike to prove ergogenic for aerobic-oriented exercises in trained and untrained individuals”. The hypothesis for the ergogenic effect of Q on aerobic performance originated from its ability to increase the mitochondrial biogenesis found in mice [22]. The reasons why many authors failed to find any ergogenic effect may be connected to the fact that endurance capacity is limited, to a greater extent, by oxygen delivery via the cardiovascular system and that the augmented mitochondrial biogenesis facilitated by Q has been found in animal models [22] but not in humans [23]. Moreover, it seems valuable to also investigate the ergogenic potential of Q supplementation in strength exercises. In fact, Q has been shown to affect the Ca^2+^ release from the sarcoplasmic reticulum (SR) [24], which can be increased in a caffeine-like fashion as a result of the Q inhibitory effect on the Ca^2+^ ATPase as well as its stimulatory effect on Ca^2+^ release channels [25]. Lee and colleagues also demonstrated that Q exerts a considerably stronger agonist action than caffeine on single Ca^2+^ release channel activity. The increased sensitivity of myofibrils to Ca^2+^ could positively affect muscle strength, as already demonstrated after caffeine supplementation [11]. Moreover, since it has been suggested that, like caffeine, Q may have a blocking effect on the adenosine receptors at a central level, this may be also involved in the energetics of neurotransmitter uptake [22] and may influence motor unit recruitment capacity. The Q-mediated augmented Ca^2+^ availability and improved recruitment capacity may explain the significant increase in the isometric strength found in PRE compared to BASE and in Q with respect to PLA. 

Concerning the potential role of Q in protecting the skeletal muscle from EEIMD, our data suggest that the neuromuscular system may be sensitive to Q supplementation. In fact, in the present study, the elbow flexor strength decay that occurred inevitably during the eccentric protocol was reduced when participants consumed Q compared to PLA. Moreover, the strength loss recorded POST-exercise in both isometric and isokinetic contractions was significantly lower with Q supplementation. It has been proven that muscular activity promotes oxidant production in contracting muscle fibers, which has been shown to play an important physiological role in the regulation of muscle force production and contraction-induced adaptive responses to exercise training [26]. In particular, it has been shown that although a small increase in ROS in muscle fibers may promote an increase in force, high ROS concentrations are associated with a decrease in force, as proposed by Reid and colleagues in a theoretical model describing a dose-dependent relationship [27]. Nevertheless, very few studies have focused on the influence of Q on muscle force production. The impairment of skeletal muscle function in terms of its force-generation capacity has been shown to be the most reliable and valid marker of the magnitude of muscle damage [28]. In fact, studies on the etiology of EEIMD have proposed that the initial phase may be caused by excessive stretching and rupture of myofibril filaments or failure in the excitation–contraction coupling system. Independently of the sequence of events following this phase (i.e., inflammatory and oxidative responses), muscle weakness is the most immediate functional consequence of EEIMD. A greater muscle weakness may indicate more extensive myofibrillar disruption.

Recently, the problem of the efficacy of dietary antioxidant supplementation for physical exercise has become an important and highly debated topic. Some studies using high doses of antioxidants (vitamins E and C) have shown that such supplementation may counteract the up-regulation of endogenous antioxidant defenses that are naturally evoked in response to exercise [29,30]. Nevertheless, several studies have reported that supplementation with Q diminishes exercise-induced oxidative stress and decreases inflammatory markers [7,31,32,33].

In the present study, even if we did not perform mechanistic experiments to explain the attenuated weakness found after Q supplementation, we can speculate that Q, being a lipophilic compound, may cross membranes easily and promote membrane stability by its radical-scavenging activities [7,34]. Indeed, it has been reported that Q, having several phenolic −OH groups, is a powerful in vitro free radical scavenger [35,36]. It is noteworthy that in isolated rat liver mitochondria, Q quenched superoxide radicals and attenuated Fe^2+^/citrate-derived membrane lipid peroxidation [37,38].

It is worth mentioning that the beneficial effects of Q largely depend on its bioavailability after oral administration. In humans, Q can be detected in plasma within 15–30 min of ingestion of a 250 or 500 mg Q chew preparation, reaching a peak concentration at approximately 120–180 min and returning to baseline levels at 24 h [39,40]. Interestingly, it has been demonstrated that a dose of 500 mg of Q aglycone supplied in tablets is comparable with the Q present in approximately 100 g of fresh red onion, as assessed by urinary excretion [41]. Hence, the present investigation was conducted over a period of two weeks with two daily intakes of Q (500 mg every 12 hours) based on both real food composition and the bioavailability test results already reported in healthy volunteers [41].

The effect of Q supplementation on muscle function could allow better maintenance of contractile function of a higher number of fibers, particularly the fast type glycolytic fibers, which are the ones that are mainly damaged during eccentric contractions [42]. Accordingly, we also found higher MFCV values in Q during the isokinetic contractions performed after the eccentric exercise. Since volunteers were requested to exert maximal force, ideally, all available motor units were recruited and the maximum firing frequency was achieved [43]. However, exercise-induced ultrastructural damage reduced the quantity and quality of available motor units, as evidenced by a lower MFCV and higher strength loss after EEIMD in the FV relationship under PLA condition. The MFCV decrease is generally explained as increased inhibition or removal of fast motor units from the contraction. From this, it appears that eccentric exercise induces greater fast motor unit injury [44], and Q may protect the muscle fiber membranes from damage. Considering this, the quantity (1 g/day) and the duration (2 weeks) of Q supplementation used in this study may have protected and stabilized membranes in the myocytes and preserved excitation–contraction coupling immediately POST-exercise, an effect that may have reduced the muscle damage and attenuated the weakness immediately after exercise. 

When muscle fibers are severely damaged and plasma membrane is disrupted, muscle fibers become necrotic, and soluble proteins leak out from the plasma membrane and get into the bloodstream [45]. In the present study, as expected, we did not find elevated CK and LDH levels in the POST-session for both Q and PLA conditions. This is not surprising, since the delayed peak of the above-mentioned biochemical markers is well known. For this reason, we also assessed their plasma concentrations in the days after the EEIMD, and we found that when participants ingested Q, the increase in both parameters was lower compared to PLA. This finding is in line with the hypothesis of Q’s role in protecting muscle fibers from damage [7,46], so the extent of membrane disruption may have been lower following Q, in turn, attenuating the increases in CK and LDH. Moreover, in the POST-session, the relaxed elbow angle was less flexed in Q compared to PLA, which could also be interpreted as a sign of a greater damage in this last condition.

## 5. Conclusions

Taken together, the findings from the present study suggest that quercetin can attenuate the severity of muscle weakness caused by eccentric-induced myofibrillar disruption and sarcolemmal action potential propagation impairment. Although the exact mechanism is unknown, Q, as a lipophilic compound, may be able to cross membranes easily and promote membrane stability, preserving the excitation–contraction coupling in myocytes [34]. In conclusion, Q seems to be a suitable nutritional supplement to reduce symptoms of discomfort and strength loss which follow an intense bout of eccentric exercise and may represent a strategy to improve overall physical fitness and, possibly, to enhance performance and training.

## Figures and Tables

**Table 1:** Mean MFCV values obtained during the force–velocity (FV) relationship task in Q and PLA conditions at baseline (BASE), before (PRE) and after (POST) the eccentric exercise. Data are expressed as a percentage of MVICBASE.

		MFCV (%)				
		0 deg/s	30 deg/s	60 deg/s	120 deg/s	240 deg/s
**Baseline**	**Q**	100	97.7 ± 1.7	100.3 ± 1.8	103.2 ± 2.0	105.8 ± 2.2
	**PLA**	100	91.7 ± 1.9	94.7 ± 1.6	96.9 ± 1.8	99.6 ± 2.2
**PRE**	**Q**	102.4 ± 1.8	98.7 ± 1.8	102.3 ± 1.8	105.6 ± 2.3	109.1 ± 2.3
	**PLA**	100.2 ± 1.4	93.6 ± 1.7	96.5 ± 1.6	98.7 ± 1.6	101.4 ± 1.8
**POST**	**Q**	100.5 ± 1.9	96.4 ± 2.1	98.9 ± 2.2	101.7 ± 2.3	105.1 ± 2.6
	**PLA**	89.8 ± 2.1 ^#§^	89.8 ± 1.9	92.2 ± 1.6	87.4 ± 2.7 ^#§^*	87.0 ± 2.2^#§^*

#, §, * *p* < 0.05; significantly different from BASE, PRE and Q respectively.

**Table 2:** Mean absolute values of arm circumference, relaxed elbow angle and visual analogue scale (VAS) recorded at baseline (BASE), before (PRE) and after (POST) the eccentric exercise in Q and PLA conditions.

		Arm Circumference	Elbow Angle	VAS
		cm	deg	cm
**Baseline**	**Q**	31.1 ± 0.7	175.4 ± 1.4	0
	**PLA**	30.6 ± 0.7	175.0 ± 1.9	0
**PRE**	**Q**	31.1 ± 0.7	174.9 ± 1.4	0
	**PLA**	30.7 ± 0.8	175.1 ± 1.5	0
**POST**	**Q**	32.2 ± 0.7 ^#§^	167.1 ± 1.8 ^#§^	0.4 ± 0.2
	**PLA**	32.2 ± 0.8 ^#§^	161.6 ± 2.3 ^#§*^	1.2 ± 0.4 ^#§^

#, §, * *p* < 0.05; significantly different from BASE, PRE and Q respectively.

**Table 3:** Mean plasma creatine kinase (CK) and lactate dehydrogenase (LDH) values measured in Q and PLA conditions at baseline (BASE), before (PRE), immediately (POST), 24 h, 48 h and 72 h after the eccentric exercise.

		CK	LDH
		a.u.	a.u.
**Baseline**	**Q**	1	1
	**PLA**	1	1
**PRE**	**Q**	0.98 ± 0.11	0.99 ± 0.05
	**PLA**	1.18 ± 0.19	1.11 ± 0.07
**POST**	**Q**	0.91 ± 0.08	1.04 ± 0.06
	**PLA**	1.22 ± 0.18	1.20 ± 0.09
**24 h**	**Q**	1.15 ± 0.14	1.07 ± 0.04
	**PLA**	1.73 ± 0.28	1.33 ± 0.08 *
**48 h**	**Q**	1.20 ± 0.17	1.26 ± 0.06
	**PLA**	2.54 ± 0.49 *	1.44 ± 0.07 *
**72 h**	**Q**	1.26 ± 0.15	1.23 ± 0.06
	**PLA**	5.89 ± 0.58 *	1.49 ± 0.09 *

a.u.: arbitrary unit; * *p* < 0.05; significantly different from Q.
